# Differential Association of Uncoupling Protein 2 Polymorphisms with Pattern Identification among Korean Stroke Patients: A Diagnostic System in Traditional Korean Medicine

**DOI:** 10.1155/2012/532078

**Published:** 2012-08-13

**Authors:** Ji Hye Lim, Mi Mi Ko, Hoyoung Lee, Ho Yeon Go, Tae-Woong Moon, Min Ho Cha, Myeong Soo Lee

**Affiliations:** ^1^Medical Research Division, Korea Institute of Oriental Medicine, 1672 Yuseongdae-ro, Yuseong-gu, Daejeon 305-811, Republic of Korea; ^2^Department of Korean Oriental Medicine, Semyung University, 836 Bongbang-dong, Chungju 380-960, Republic of Korea

## Abstract

Uncoupling protein 2 (UCP2), a mitochondrial protein present in many organs and cell types, is known to dissipate the proton gradient formed by the electron transport chain. Its function is correlated with predictive parameters, such as obesity, diabetes, and metabolic syndromes. We analyzed the distribution of UCP2 polymorphisms in stroke patients diagnosed with one of the following four stroke subtypes based on the TKM standard pattern identification (PI): Qi-deficiency (QD), Dampness and Phlegm (D&P), Yin-deficiency (YD), and Fire and Heat (F&D). We studied a total of 1,786 stroke patients (397/QD, 645/D&P, 223/YD, and 522/F&D, 586/normal). Genotyping for the G-1957A, G-866A and A55V UCP2 polymorphisms was performed using the TaqMan. G-866A and A55V were significantly associated with the D&P and H&F subtypes. The frequency of subjects with the A allele of G-866A was significantly lower than the frequency of subjects with the GG type. The A55V polymorphism was also shown similar effect with G-866A in the dominant model. In contrast, no SNPs were shown to be associated with the QD or YD subtypes in this study. These results showed that the G-866A and A55V UCP2 polymorphisms may be genetic factors for specific PI types among Korean stroke patients.

## 1. Introduction

The uncoupling proteins (UCPs) are a protein family comprised of mitochondrial proteins located at the inner membrane of mitochondria [1]. They function in the dissipation of proton gradients formed by the electron transport system and play roles in the homeostasis of body temperature by thermogenesis and in decreasing ROS production [2, 3]. The UCP family is composed of five subtypes: UCP1, UCP2, UCP3, UCP4, and UCP5. UCP1 and UCP3, which both function in thermogenesis, have tissue-specific expression patterns. The major sites of UCP1 and UCP3 expression are brown adipose tissue and muscle, respectively [1, 4]. Unlike UCP1 and UCP3, UCP2 is widely expressed in all tissues and reduces reactive oxygen species (ROS) production [3]. ROS have important roles in cell signaling and homeostasis [5, 6], but increase of ROS level by environmental stress and cellular metabolism causes abnormal inflammatory responses, atherosclerosis and cardiovascular disease [7–11], and UCP2 is involved in the preservation of ROS homeostasis and is associated with many cardiovascular diseases.

The UCP2 gene is located at chromosome 11q13, and many studies have reported the association of the G-866A and A55V polymorphisms of UCP2 with various diseases, such as obesity, diabetes, and metabolic syndromes [2, 12–15]. For example, A55V was significantly associated with obesity in men of Japanese. The frequency of subjects with the AV or VV types in the obese group was 67.4%, which was significantly lower than the frequency observed in the nonobese group (78.1%) [14]. Another report showed that G-866A was associated with a reduction in the prevalence of type 1 diabetes in Germany [15]. These data suggest that polymorphisms of UCP2 are associated with obesity and its related metabolic syndromes and diseases.

In traditional Korean medicine (TKM), subjects with disease are diagnosed according to pattern identification (PI). PI is a comprehensive system for the diagnosis of disease according to the subject's signs and symptoms. The signs and symptoms reflect a personal state; thus, PI is a diagnostic system for the cause, nature, and location of the illness, the patient's physical condition, and the patient's treatment [16–21]. TKM classifies stroke into four sub-PI groups: Qi-deficiency (QD), Dampness and Phlegm (D&P), Yin-deficiency (YD), and Fire and Heat (F&D) [22]. Among the PI subtypes defined as follows according to Min et al. [23], the D&P is characterized by its impediment to Qi movement, turbidity, heaviness, stickiness, and downward flowing properties and shows an eminent tendency toward obesity and increase of serum lipids. The H&F accounts for pathogenic fire characterized by intense heat that is apt to injure fluid and to consume Qi. The YD indicates pathologic change marked by deficiency of Yin and being known to highly contribute to emaciation, rarely to obesity. The QD is a deficiency of qi that leads to decreased visceral function and lowered body resistance. 

PI is affected by hereditary factor as well as environmental factors. Some reports showed relationships between genetic polymorphisms and PI in Chinese, Korean, and Japanese populations [24–27]. Finding genes related with stroke-PI, we previously reported that NPY and PON1 polymorphism wass associated with the D&P [24, 28]. Because UCP2 is involved in cellular metabolism and thermogenesis, and some SNPs in UCP2 were known to be associated with obese/overweight and serum lipid, we hypothesized that SNPs, changing UCP2 expression or amino acid, might be related with PI subtypes including the F&H related with thermogenesis and the D&P, YD related with metabolism imbalance. In this study, we investigated the G-1987A, G-866A, and A55V UCP2 polymorphisms in 586 normal subjects and 1,786 stroke patients diagnosed by two expert TKM doctors using PI, and we thereby elucidated the association of UCP2 polymorphisms with PI.

## 2. Subjects and Methods

### 2.1. Subjects

We examined 1,786 subjects diagnosed with stroke and 586 normal subjects without stroke. The stroke patients were admitted into the twelve oriental medical hospitals participating in this study; these hospitals are located throughout South Korea to minimize regional differences: KyungHee Oriental Medical Center (Seoul), KyungHee East-West Neo Medical Center (Seoul), DongGuk International Hospital (Kyunggi-do), Kyungwon Oriental Medical Hospitals (Seoul and Inchon), DongSeo Oriental Medical Hospital (Seoul), DaeJeon Oriental Medical Hospital (Daejeon), DongSin Oriental Medical Hospital (Gwangju and Jeollanam-do), Wonkwang Oriental Medical Hospital (Jeollabuk-do), Woo Suk Oriental Medical Hospital (Jeollabuk-do), and SangJi Oriental Medical Hospital (Gangwon-do). Some of the included subjects overlapped with our previous study [24]. The stroke diagnosis was confirmed by brain computer tomography (CT), magnetic resonance imaging (MRI), or magnetic resonance angiography (MRA), and the clinical data from the subjects were collected after obtaining written informed consent. The normal subjects were recruited from DaeJeon Oriental Medical Hospital and Wonkwang Oriental Medical Hospital, and stroke status was confirmed by MRI. This study was performed after receiving approval by the Institutional Review Boards of the Korean Institute of Oriental Medicine and by each of the Oriental Medical Hospitals.

### 2.2. PI Diagnosis of Stroke Patients

The PI diagnosis of subjects was performed as described in the report published by Lee et al. [22]. Briefly, the signs and symptoms presented by the patients were classified using the “Stroke PI case report form” [16]. The PI diagnosis of each patient was determined by two expert TKM doctors, and subjects receiving differing diagnoses from the two doctors were excluded. The number of patients in each PI group was as follows: 397 in the QD group, 645 in the D&P group, 223 in the YD group, and 522 in the H&D group.

### 2.3. Genomic DNA Preparation and Genotyping

Genomic DNA from each subject was prepared from blood using the GeneAll genomic DNA extraction kit (GeneAll, Seoul, Korea). The UCP2 single-nucleotide polymorphisms (SNPs) analyzed in this study are listed in Table 2. The genotyping of all analyzed SNPs was performed by Macrogen Inc. (Seoul, Korea) using the polymerization chain reaction (PCR) with TaqMan probes. To confirm the accuracy of the genotype analysis, five percent of our subjects were randomly selected, and their SNPs were genotyped by the sequencing method using the ABI 3700 sequencer (ABI Inc. Carlsbad, CA USA). The SNP-genotyping agreement between the two methods was 97.95%, which represents high accuracy. The concordance of UCP2 SNPs in normal with Hardy-Weinberg equilibrium (HWE) was tested using HapAnalyzer software [29].

### 2.4. Statistical Analysis

The statistical analysis of our data was performed with SAS software version 9.1.3 (SAS Institute Inc., Cary, NC USA). Differences in continuous variables were determined by nonparametric (Wilcoxon rank-sum test) tests. Categorical variables were compared with a chi-squared test or Fisher's exact test. A multiple logistic regression adjusted for age, sex, smoking status, and drinking status was performed to estimate the association of SNPs with the normal group versus each of the PI groups, and odds ratios (ORs) with 95% confidence intervals (95% CI) were obtained. We performed a statistical analysis using a general linear model adjusted for age, sex, and smoking status to investigate whether the PIs are associated with the serum parameters of the patient subjects. The statistical significance was set at *P* < 0.05.

## 3. Results

**Figure 1:**
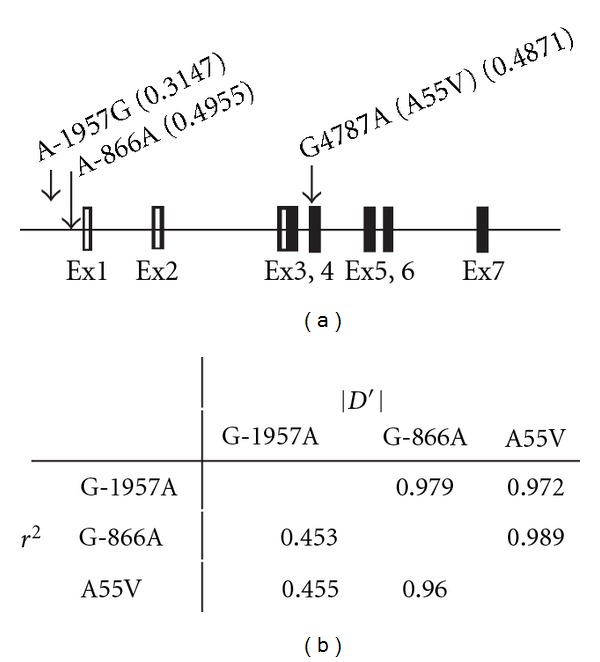
(a) Map of the SNPs located in the UCP2 gene. The values in parentheses show the minor allele frequency. The open and closed boxes in the exons indicate the untranslated and translated regions, respectively. (b) Linkage distribution among three SNPs.

Table 1 shows the clinical differences between each of the PI groups and the normal group. The history of disease and the serum parameters of each group displayed a pattern similar to that of our previous reports [24, 25]. However, obese indices, such as BMI and waist circumference, showed a significantly different pattern in the PI groups compared with the normal group. The median BMIs of the QD and YD groups were 22.6 kg/m^2^ and 22.5 kg/m^2^, respectively, which were significantly lower than the 24.2 kg/m^2^ BMI of the normal subjects (*P* < 0.001). In contrast, the BMIs of the D&P and H&F groups were not different compared to the normal group (*P* = 0.056 and *P* = 0.120, resp.). Waist circumference showed the opposite pattern; it was significantly increased in the D&P and H&F groups, but not in the QD and YD groups, compared to the normal group. Differences in the body characteristics and serum lipid parameters among the PI groups were shown in supplemental available online at doi:10.1155/2012/532078. The mean of obese indices such as BMI, waist circumference and WHR were significantly higher in the D&P and H&F groups than YD and Qi groups, and the level of triglycerides in the D&P group was also higher in the D&P group than that of the other three groups. The location of three SNPs within the UCP2 gene is shown in Figure 1(a), and their linkage distribution among three SNPs in Figure 1(b).

The characteristics of the SNPs investigated in this study are listed in Table 2. Two of the SNPs are located at the −1957 and −866 positions relative to the transcriptional start site. The other SNP is situated at amino acid 55 in UCP2 exon 4, which causes the change of an alanine to a valine. All of the alleles were in Hardy-Weinberg equilibrium (*P* > 0.05) in the normal group, according to the recommended International HapMap Project guidelines. An analysis of the linkage distribution among the three SNPs indicated that G-866A and A55V were tightly linked. The *D*′ and *r* ^2^ values were 1.00 and 0.97, respectively, which are similar to those reported in a previous report by Cha et al. [30].

Table 3 shows the SNP distribution in each PI group compared to the normal group. G-866A was significantly associated with D&P and H&F. The ratio of subjects with the A allele was 69.58% in the D&P group and 71.92% in the H&F group, both of which were significantly lower than the 76.66% ratio of the normal group, after adjusting for sex, age, smoking, and drinking (OR = 0.668 (0.501–0.890), *P* = 0.006; OR = 0.647 (0.472–0.886), *P* = 0.007, resp.). The A55V SNP, which is tightly linked with G-866A, showed similar pattern of G-866A in the dominant model. The frequency of A allele of A55V is 68.97% in the D&P group and 70.27% in the H&F group, which are significantly lower than the 75.6% ratio of the normal group (*P* = 0.010 in D&P, *P* = 0.007 in H&F). In contrast, no SNPs were associated with QD or YD in this study.

To confirm which factor was affected by G-866A or A55V, we compared serum lipid parameters according to genotype in normal subjects in Table 4. A55V was associated with serum triglyceride. The mean serum triglyceride of the subjects with the AV and VV type at the 55 position of the UCP2 gene was 142.6 mg/dL and 148.82 mg/dL, respectively. These means were significantly lower than the mean serum triglyceride (158.58 mg/dL) of the subjects with the AA type in the dominant mode (*P* = 0.0471).

## 4. Discussion

Stroke is the second most common cause of death in Korea; 52 women and 50.3 men per one hundred thousand people died because of stroke in 2009 [31]. Therefore, many efforts to prevent the occurrence of stroke are necessary. Additionally, the rapid and appropriate treatment of stroke is also important to remove or reduce the aftereffects of a stroke. Recently, personalized medicine has been emphasized; in this model, instead of the uniform treatment of disease, patients receive different treatments according to the patient's individual condition, which might increase the positive effects of treatment and reduce the adverse effects [32–34]. In TKM, patients with the same disease are given different treatments depending on the patient's condition. PI, a basic system for diagnosis in TKM, entails a systematic analysis of the patient's physical condition. Based on PI, TKM doctors determine the cause, nature, and treatment of the illness [16]. Because of these shared aspects, treatments based on PI might approximate personalized medicine, and some recent studies have been undertaken to identify genetic variation associations with PI [24, 27].

In TKM, stroke is classified into four standard subtypes according to PI: Qi-deficiency, Dampness and phlegm, Yin-deficiency, and Fire and Heat [22]. D&P is correlated with obesity and obesity-related metabolic syndromes [23, 24]. For example, Min et al. reported that total cholesterol, BMI, and waist circumference were significantly increased in D&P pattern patients [23], and this finding is similar to our results (Table 1). We also found that the clinical characteristics of the H&F pattern were similar to the D&P pattern. 

In the current study, we investigated the association of UCP2 polymorphisms with PI. UCP2 is a mitochondrial protein and is known to dissipate the proton gradient formed by electron transport and decrease ROS production [2]. The G-866A polymorphism located at the promoter region and the A55V polymorphism in exon 4 of the UCP2 gene were associated with thermogenesis and energy consumption in lipids metabolism, resulting in weight loss and low serum lipid level in Korean population studies [34, 35]. In this study, the G-866A SNP had a negative effect on the D&P and H&F patterns. A55V, which is a nonsynonymous SNP and is linked with G-866A, also showed a negative association with the D&P and H&F patterns (Table 3). Obesity indices, such as BMI, WHR, and serum lipids, showed an increase in the D&P and H&F patterns compared with the QD and YD patterns (Table 4). These results might suggest that the D&P and H&F patterns are related to obesity or that obese subjects have a lower ratio of GA and AA genotypes of the G-866A SNP. This result matched that of a previous study reported by Jun et al. [36]. In the report, G-866A and A55V were associated with a decrease of BMI and waist circumference in Korean children [36]. Other reports studying other populations also showed negative effects of G-866A on obesity [12, 14]. However, our result was in contrast to other studies reported by Wang et al. and Martinez-Hervas et al. [37, 38], who showed an increased susceptibility to obesity for two SNPs in Taiwanese and southern European populations. 

In TKM, the PI is classified by position, internal or external etiology, or organ. The D&P and H&F patterns are classified by etiology. D&P is a combination of phlegm and internal dampness that causes disease. D&P produces anorexia, dyspepsia, dizziness, headache, heaviness, and other maladies. H&F is any pattern or syndrome of heat and fire, either contracted externally or engendered internally. H&F produces hot flashes, mouth or tongue dryness, ophthalmoxerosis, and other ailments of the body [39, 40]. In TKM, D&P and F&H could be explained as causation in the pathogenesis. When damp is congested for a long time and phlegm arises from the body, damp and phlegm are mixed up each other. The longer damp and phlegm stay in the body, the more heat they generate. What is worse, D&P are combined with the heat that they generate and start to produce more complicated symptoms attributed to the mixture of damp, phlegm, and heat [41]. In addition, the F&H seems to induce so excessive heat generation and high-energy consumption that it can result in the obesity by increasing the dietary intake to supplement the energy dearth.

In this study, we showed an association of UCP2 polymorphisms with PI in Korean stroke patients. UCP2 polymorphisms, far involved in metabolism and thermogenesis, were significantly distributed to D&P and F&H, indicating that the F&H along with the D&P was another obese factor. However, this study had some limitations that prevent generalizing these results. The first limitation is the sample size disparity between the test and control group that may have impacted finding. Secondly, the PI was limited to stroke diagnoses. The lack of evidence on the accuracy of PI forced us to perform an observational study, not allowing a randomized controlled trial. Thus, further studies with greater balanced sample size that focus on other diseases will be necessary to confirm the scientific basis of the relationship between genetic variations and PI. 

## Supplementary Material

The results showed that body characteristics (weight, BMI, waist circumference, WHR) and serum lipid parameter (triglyceride) were significantly different among the four PI groups. Generally, the means of those levels in D&P and F&H groups were higher than those in QD and YD groups.

## Figures and Tables

**Table 1:** Clinical differences among the pattern identification groups of Korean stroke patients.

Characteristics	Control	Qi deficiency	*P* value	Dampness & Phlegm	*P* value	Yin deficiency	*P* value	Fire & Heat	*P* value
*N*	588	397		645		223		521	
Anthropometric characteristics									
Sex (M/F)	273/314^∗^	135/262	<.0001	314/331	0.445	97/126	0.4424	387/135	<.0001
Age (year)	64 (58, 69)^#^	69 (60, 77)	<.0001	69 (61, 75)	<.0001	73 (65, 79)	<.0001	68 (59, 75)	<.0001
Smoking (none/stop/active)	299/232/56	278/51/68	<.0001	396/95/153	<.0001	144/46/33	<.0001	202/129/191	<.0001
Drinking (none/stop/active)	357/35/195	260/37/100	0.0084	381/66/196	0.021	137/25/61	0.0205	186/81/255	<.0001
Weight (kg)	62.0 (56.0, 69.0)	56.6 (50.0, 62.8)	<.0001	70.0 (63.0, 78.0)	0.046	56.1 (50.0, 64.0)	<.0001	65.0 (57.0, 72.0)	0.0012
BMI (kg/m^2^)	24.2 (22.6, 26.0)	22.6 (20.9, 24.4)	<.0001	24.6 (22.5, 26.7)	0.059	22.5 (20.5, 24.7)	<.0001	24.0 (22.0, 26.1)	0.12
Waist circumference (cm)	85.0 (79.3, 90.0)	85.0 (79.0, 91.0)	0.436	89.0 (82.0, 95.0)	<.0001	83.0 (76.0, 89.0)	0.042	88.0 (82.0, 94.0)	<.0001
WHR	0.88 (0.84, 0.91)	0.92 (0.88, 0.96)	<.0001	0.94 (0.90, 0.97)	<.0001	0.93 (0.89, 0.96)	<.0001	0.94 (0.90, 0.98)	<.0001
History									
TIA (Yes)	15	30	0.0002	53	<.0001	26	<.0001	42	<.0001
Hypertension (Yes)	107	232	<.0001	416	<.0001	131	<.0001	325	<.0001
Hyperlipidemia (Yes)	70	50	0.7163	96	0.1242	19	0.1689	55	0.5114
Diabetes (Yes)	40	103	<.0001	187	<.0001	57	<.0001	143	<.0001
Heart disease (Yes)	0	23	<.0001	48	<.0001	14	<.0001	27	<.0001
Serum parameter									
GOP (U/mL)	23.0 (20.0, 29.0)	22.0 (18.0, 28.0)	<.0001	23.0 (18.0, 28.1)	0.0005	22.0 (18.0, 28.0)	0.0009	23.0 (18.0, 31.0)	0.1316
GPT (U/mL)	21.0 (16.0, 30.0)	18.0 (13.0, 28.0)	<.0001	19.0 (14.0, 28.0)	<.0001	17.0 (13.0, 26.0)	<.0001	21.0 (15.0, 32.0)	0.4774
Total cholesterol (mg/dL)	199.0 (175.0, 226.0)	178.0 (154.0, 211.0)	<.0001	189.0 (160.0, 219.0)	<.0001	185.0 (159.0, 212.0)	<.0001	178.0 (148.0, 207.0)	<.0001
Triglyceride (mg/dL)	131.0 (91.0, 182.0)	121.0 (88.0, 175.6)	0.398	132.0 (94.0, 195.0)	0.1055	123.5 (83.0, 174.0)	0.2128	131.5 (93.0, 194.0)	0.3019
HDL-cholesterol (mg/dL)	51.0 (43.2, 59.9)	42.9 (35.9, 52.0)	<.0001	41.0 (33.5, 48.8)	<.0001	42.7 (35.1, 49.4)	<.0001	41.0 (34.0, 49.0)	<.0001
Blood sugar (mg/dL)	99.0 (92.0, 106.0)	101.5 (91.0, 130.0)	0.0023	107.0 (94.0, 128.0)	<.0001	107.0 (98.0, 141.0)	<.0001	105.0 (94.0, 128.5)	<.0001

^∗^Indicates number of subjects, and ^#^indicates value of median (25% and 75% interquartiles). The *P* value of categorized variables was calculated by a chi-squared test or Fisher's test, and continuous values were analyzed by a Mann-Whitney *U* test after a normality test and compared with the normal group.

**Table 2:** List of the SNPs analyzed in this study.

SNP	rs no.	Position	Location relative to transcription start site	Nucleotide (amino acid) change	Location relative to p-terminus of chromosome	MAF	HWEp
G-1957A	rs649446	Promoter	−1957	G/A	73695845	0.3147	0.138
G-866A	rs659366	Promoter	−866	G/A	73694754	0.4955	0.051
A55V	rs660339	exon 2	4787	G/A (A/V)	73689104	0.4871	0.06

**Table 3:** Allele and genotype distribution of the SNPs between the control group and each PI group.

Allele															
	SNP	Allele	Normal	QD	OR (952 CI)	*P*	D&P	OR (952 CI)	*P*	YD	OR (952 CI)	*P*	F&H	OR (952 CI)	*P*
	G-1957A	G	784 (68.53)	543 (68.73)	0.983	0.8738	910 (70.87)	0.881	0.193	306 (68.92)	0.885	0.367	748 (72.06)	0.819	0.0628
		A	360 (31.47)	247 (31.27)	(0.793–1.218)		374 (29.13)	(0.727–1.066)		138 (31.08)	(0.678–1.154)		290 (27.94)	(0.663–1.011)	
	G-866A	G	561 (50.45)	408 (51.78)	0.970	0.7647	689 (53.74)	0.866	0.1129	220 (49.33)	1.039	0.7603	552 (53.08)	0.870	0.1577
		A	551(49.55)	380 (48.22)	(0.794–1.185)		593 (46.26)	(0.726–1.034)		226 (50.67)	(0.813–1.328)		488 (46.92)	(0.717–1.055)	
	A55V	G	598 (51.29)	409 (51.90)	0.967	0.7451	691 (54.15)	0.874	0.1306	227 (50.90)	1.033	0.7959	561 (54.15)	0.866	0.1418
		A	568 (48.71)	379 (48.10)	(0.793–1.178)		585 (45.85)	(0.733–1.041)		219 (49.10)	(0.809–1.317)		475 (45.85)	(0.715–1.049)	

Genotype															
Model	SNP	Genotype	Normal	QD	OR (952 CI)	*P*	D&P	OR (952 CI)	*P*	YD	OR (952 CI)	*P*	F&H	OR (952 CI)	*P*

	G-1957A	GG	261 (45.6)	191 (48.42)	0.892	0.426	321 (50.00)	0.824	0.131	110 (49.55)	0.757	0.116	270 (52.02)	0.702	**0.012**
		GA + AA	311 (54.4)	204 (51.62)	(0.672–1.183)		321 (50.00)	(0.642–1.059)		112 (50.45)	(0.534–1.071)		249 (47.98)	(0.534–0.924)	
Do	G-866A	GG	130 (23.4)	107 (27.22)	0.826	0.249	195 (30.42)	0.668	**0.006**	58 (26.01)	0.817	0.324	146 (28.08)	0.647	**0.007**
		GA + AA	426 (76.6)	287 (72.82)	(0.596–1.144)		446 (69.58)	(0.501–0.890)		165 (73.99)	(0.548–1.220)		374 (71.92)	(0.472–0.886)	
	A55V	GG	142 (24.4)	109 (27.72)	0.814	0.207	198 (31.03)	0.694	**0.01**	62 (27.80)	0.771	0.194	154 (29.73)	0.653	**0.007**
		GA + AA	441 (75.6)	285 (72.32)	(0.591–1.121)		440 (68.97)	(0.525–0.918)		161 (72.20)	(0.522–1.141)		364 (70.27)	(0.480–0.888)	

	G-1957A	GG + GA	523 (91.4)	352 (89.12)	1.273	0.32	589 (91.74)	0.927	0.738	196 (88.29)	1.213	0.513	478 (92.10)	1.049	0.847
		AA	49 (8.6)	43 (10.92)	(0.791–2.05)		53 (8.26)	(0.595–1.444)		26 (11.71)	(0.680–2.166)		41 (7.90)	(0.644–1.710)	
Re	G-866A	GG + GA	431 (77.5)	301 (76.42)	1.125	0.493	494 (77.07)	1.038	0.805	162 (72.65)	1.359	0.134	406 (78.08)	1.075	0.662
		AA	125 (22.5)	93 (23.62)	(0.804–1.575)		147 (22.93)	(0.770–1.401)		61 (27.35)	(0.910–2.030)		114 (21.92)	(0.777–1.489)	
	A55V	GG+GA	456 (78.2)	300 (76.12)	1.135	0.455	493 (77.27)	1.029	0.853	165 (73.99)	1.204	0.367	407 (78.57)	0.937	0.695
		AA	127 (21.8)	94 (23.92)	(0.814–1.584)		145 (22.73)	(0.764–1.385)		58 (26.01)	(0.805–1.801)		111 (21.43)	(0.677–1.297)	

Odd ratios and *P* values were calculated by binary logistic regression model after adjustment for sex, age, smoking, and drinking and compared to the normal group.

**Table 4:** Association analysis of serum biochemical parameters by genotype of UCP2 in normal group.

Parameter	Genotype						*P* value			
	G-866A			A55V			G-866A		A55V	
	GG	GA	AA	GG	GA	AA	Do	Re	Do	Re
*N*	130	302	125	142	315	127				
Body characteristics										
Weight (kg)	61.78 ± 8.57	62.78 ± 9.64	63.29 ± 8.87	61.72 ± 8.34	62.77 ± 9.84	62.9 ± 8.99	0.9198	0.8045	0.966	0.9862
BMI (kg/m^2^)	24.5 ± 2.56	24.4 ± 2.86	24.31 ± 2.43	24.49 ± 2.51	24.41 ± 2.9	24.27 ± 2.48	0.5930	0.7802	0.5594	0.5889
Waist circumference (cm)	83.82 ± 7.58	84.7 ± 8.39	87.44 ± 30.33	83.84 ± 7.39	84.69 ± 8.49	87.25 ± 30.16	0.6955	0.0888	0.7126	0.1086
WHR	0.87 ± 0.09	0.88 ± 0.05	0.9 ± 0.3	0.87 ± 0.09	0.88 ± 0.05	0.9 ± 0.29	0.5733	0.1024	0.5423	0.108
Serum lipid parameters										
Total cholesterol (mg/dL)	204.6 ± 40.45	200.97 ± 40.35	201.2 ± 37.39	203.65 ± 39.47	200.91 ± 40.13	203.32 ± 38.77	0.4809	0.9282	0.7317	0.6097
Triglyceride (mg/dL)	155.33 ± 99.57	141.94 ± 74.52	151.14 ± 72.41	158.58 ± 104.46	142.6 ± 74.7	148.82 ± 67.27	0.1073	0.6033	**0.0471**	0.8815
HDL-cholesterol (mg/dL)	52.36 ± 12.9	52.73 ± 13.15	51.84 ± 13.02	52.68 ± 13.24	52.76 ± 13.15	52.08 ± 12.63	0.7847	0.7345	0.9756	0.6987
Atherogenic index	0.41 ± 0.30	0.39 ± 0.26	0.43 ± 0.26	0.41 ± 0.31	0.39 ± 0.26	0.43 ± 0.25	0.4996	0.2465	0.5006	0.3187

The value indicates the number of subjects (mean ± SD). The *P* value was calculated using a general linear model adjusted for sex, age, smoking, and drinking.
