# Supplementary material for: Differential Association of Uncoupling Protein 2 Polymorphisms with Pattern Identification among Korean Stroke Patients: A Diagnostic System in Traditional Korean Medicine
Source: Evid Based Complement Alternat Med. 2012 Aug 13;2012:532078. doi: 10.1155/2012/532078 (PMC3424818; doi:10.1155/2012/532078)
Supplement: Supplementary file 1 — The results showed that body characteristics (weight, BMI, waist circumference, WHR) and serum lipid parameter (triglyceride) were significantly different among the four PI groups. Generally, the means of those levels in D&P and F&H groups were higher than those in QD and YD groups. [file 532078.f1.pdf]

Supplement 1. Differences in the body characteristics and serum lipid parameters among the pattern identification groups of Korean stroke patients

Characteristics	QD	D&P	YD	F&H	p
body characteristics					
weight (kg)	57.16 ±9.3*	63.62 ±10.46	56.84 ±10.07	64.42 ±10.33	<0.001
BMI (kg/m^2)	22.87 ±2.90	24.73 ±3.22	22.65 ±3.15	24.08 ±2.92	<0.001
waist circumference (cm)	85.06 ±8.60	89.0 ±9.46	83.05 ±9.25	88.04 ±8.90 0	<0.001
WHR	0.927 ±0.100	0.943 ±0.077	0.936 ±0.142	0.948 ±0.092	<0.001
serum lipid parameters					
total cholesterol (mg/dL)	185.29 ±49.18	190.06 ±46.82	186.27 ±40.65	179.46 ±44.930	0.149
triglyceride (mg/dL)	156.06 ±148.78	164.33 ±115.24	145.47 ±95.23	157.99 ±95.43	**0.032**
HDL-cholesterol (mg/dL)	44.85 ±13.31	42.40 ±11.92	44.09 ±13.60	42.37 ±11.90	0.303
Atherogenic index	0.478 ±0.298	0.527 ±0.287	0.472 ±0.304	0.525 ±0.277	0.056

* indicates mean ± standard deviation

The p values among the PI groups were calculated by a general linear model adjusted for sex, age, smoking and drinking
